# Spin and reporting bias in the use of platelet-rich plasma for the treatment of knee osteoarthritis

**DOI:** 10.1007/s00590-025-04353-x

**Published:** 2025-08-12

**Authors:** Mary K. Richardson, Natalie M. Kistler, Cory K. Mayfield, Kevin C. Liu, Jay R. Lieberman, Nathanael D. Heckmann

**Affiliations:** Keck Medical Center of USC, Los Angeles, United States

**Keywords:** Platelet rich plasma, Knee osteoarthritis, Spin, Bias reporting

## Abstract

**Introduction:**

Systematic reviews and meta-analyses evaluating platelet-rich plasma (PRP) injections are vulnerable to spin (i.e., misrepresentation of study findings). We sought to describe the incidence of spin in the abstracts of systematic reviews and meta-analyses reporting on PRP for knee osteoarthritis.

**Methods:**

A Preferred Reporting Items for Systematic Reviews and Meta-Analyses search was conducted in Embase, PubMed, and Cochrane databases to identify systematic reviews and meta-analyses on PRP injections for knee osteoarthritis. Data collected from abstracts included 21 types of spin as originally reported by Yavchitz et al., year and journal of publication, level of evidence, and Scopus CiteScore™. Associations between the most common and severe spin types and abstract characteristics were assessed using Spearman analyses.

**Results:**

In all 79 abstracts of systematic reviews and meta-analyses assessed, at least one form of spin was identified. The average spin score per abstract was 5.46 ± 2.30 (range 1–12). The most common type of spin was “conclusion claiming the beneficial effect of the experimental treatment despite high risk of bias in primary studies” (67/79, 84.8%). There was a correlation between the spin type “authors hide or do not present any conflict of interest” and an older publication year (rho: − 0.252, *P *value: 0.025).

**Discussion:**

Spin favoring PRP is highly prevalent in the abstracts of systematic reviews and meta-analyses that assess the use of PRP for the treatment of knee osteoarthritis. This inaccurate presentation of information identifies a need for (1) high-quality RCTs and (2) reduction of spin in future literature to accurately distribute information regarding PRP for this indication.

**Supplementary Information:**

The online version contains supplementary material available at 10.1007/s00590-025-04353-x.

## Introduction

Knee osteoarthritis has become one of the fastest increasing major health conditions faced by aging Americans [1, 2]. Management of knee osteoarthritis costs an estimated $89.1 billion annually in the USA as well as associated patient morbidity such as pain and activity limitations [3, 4]. Consequently, there is an increasing demand for cost-effective and reliable non-surgical treatment modalities that alleviate symptoms associated with knee osteoarthritis, particularly for patients who are not yet eligible for total knee replacement [5, 6].

Platelet-rich plasma (PRP) injections have been increasingly used for the treatment of knee osteoarthritis; however, the data supporting its use are heterogeneous and lacking consensus regarding indication and utility [7–14]. Many published articles have demonstrated the favorable pain relief and safety profile, ease of preparation and administration, and relatively low cost of PRP preparation. As such, the PRP market is expected to increase exponentially in the next ten years [15–17]. With this considerable financial incentive, there is increasing research and pressure to publish promising results of this emerging technology. However, there is significant criticism regarding the existing clinical and scientific evidence on PRP’s efficacy, given that previous research is inconclusive and limited by heterogeneity in PRP preparation and study design [7, 16, 18–21]. The considerable revenue generated from PRP and other orthobiologic injections used for the management of knee osteoarthritis, particularly when compared to other treatment modalities such as over-the-counter anti-inflammatories and physical therapy, may incentivize significant industry and financial interest to succeed [16].

As researchers investigating the utilization of PRP for knee osteoarthritis may be incentivized to demonstrate positive findings, we sought to identify whether the literature evaluating the use of PRP for knee osteoarthritis was at increased risk of spin, defined as the reporting of data in a manner that overstates beneficial effects or understates negative effects despite insufficient evidence to support those conclusions [23–25]. Despite the available clinical practice guidelines, clinicians often make treatment decisions based on systematic reviews and meta-analyses, the primary goal of this study was to use a validated spin assessment tool to identify and categorize the incidence of spin and related bias in the abstracts of systematic reviews and meta-analyses evaluating the utilization of PRP for knee osteoarthritis [23, 26, 27]. Secondary goals included establishing correlations between various spin types and abstract characteristics, including the year of publication, and the level of evidence included.

## Methods

A Preferred Reporting Items for Systematic Reviews and Meta-Analyses (PRISMA) search was conducted in Embase, PubMed, and Cochrane databases using a predetermined protocol [28]. Search terms included, (“Meta-Analysis [Publication Type] OR systematic review[Filter] OR"systematic review"OR"systematic reviews"OR meta-analysis OR metaanalysis OR “meta analysis” OR metanalysis OR meta-analyses OR metaanalyses OR “meta analyses” OR PRISMA”) AND (“Platelet-Rich Plasma” OR “PRP” OR “Platelet Rich Plasma” OR OR “Thrombocyte-Rich Plasma” OR “Thrombocyte Rich Plasma” OR OR “plasma rich in growth factors” OR PRGF OR “platelet derived growth factor” OR “platelet derived” OR “platelet gel” OR “platelet concentrate” OR PRF OR “platelet rich fibrin” OR ACP OR “autologous conditioned plasma” OR “APS” OR “autologous protein solution” OR “platelet lysate”) AND"Osteoarthritis"[Mesh] OR “osteoarthritis” OR “OA”). Results were collected and deduplicated using EndNote 20 (Clarivate, Philadelphia, PA, USA).

### Eligibility

Two independent authors (M.K.R. and N.M.K.) reviewed publications for study eligibility. Criteria included systematic reviews or meta-analyses published in English, peer-reviewed journals on the use of PRP as a primary treatment arm for knee osteoarthritis. Studies that were not systematic reviews or meta-analyses, not published in English, lacked an accessible full-text or abstract, or included non-human or cadaver subjects were excluded. Additionally, those that evaluated non-injectable PRP or PRP as a secondary study outcome or non-primary comparative group, or utilized injected PRP in a location that was not the knee, or for any indication other than knee osteoarthritis were excluded.

### Abstract Evaluation

**Fig. 1:**
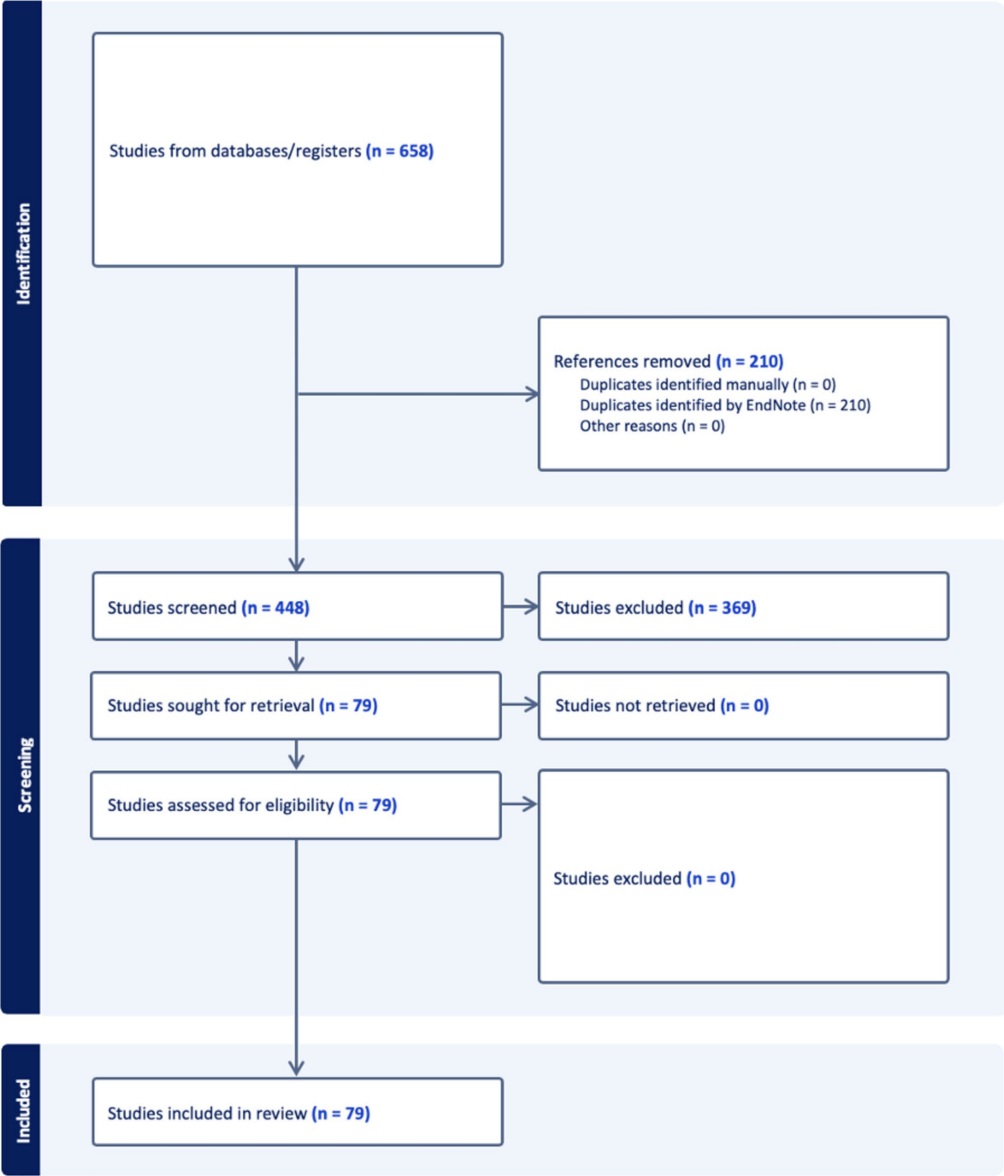
PRISMA literature screening figure

After deduplication, 448 studies were retrieved using the search terms above, 79 of which were eligible for abstract evaluation after screening (Fig. 1, Supplemental Table 1). Two reviewers (M.K.R. and N.M.K.) were trained to identify the 21 types of spin as originally reported by Yavchitz et al. [27] to evaluate the abstracts of the identified studies (Table 1). There are three categories described including “misleading reporting,” “misleading interpretation,” and “inappropriate extrapolation.” The presence of each spin type received 1 point; the absence of each spin type received 0 points. The total score possible per abstract was 21 points, with higher scores indicating increasing frequency of spin. Each reviewer independently evaluated abstracts for the presence of spin per the validated tool. Full texts were reviewed if authors disagreed or if additional clarity was required. Scoring conflicts were mitigated by a third trained reviewer (C.K.M.). The full text was evaluated to collect the year and journal of publication, Level of Evidence according to guidelines published by the American Academy of Orthopaedic Surgeons, study design, funding source, and reported adherence to PRISMA guidelines [28, 29]. We additionally assessed the Scopus CiteScore™, which is a tool offered by a leading scientific publisher to evaluate impact and influence of a given publication (Elsevier, Amsterdam, Netherlands 2016) [30].

**Table 1:** Overall spin frequency by category

Category	Description	*N*	%
Misleading reporting	Selective reporting of or overemphasis on efficacy outcomes favoring the beneficial effect of the experimental intervention	29	36.71
	Selective reporting of or overemphasis on harm outcomes favoring the safety of the experimental intervention	4	5.06
	Failure to report a wide confidence interval of estimates	57	72.15
	Authors hide or do not present any conflict of interest	46	58.23
	Inadequate focus on the results of primary studies favoring the beneficial effect of the experimental intervention instead of the meta-analysis results	10	12.66
	Conclusion focusing selectively on statistically significant efficacy outcome	25	31.65
	Failure to report the number of studies/patients actually contributing to the analysis for main outcomes	39	49.37
	Failure to specify the direction of the effect when it favors the control intervention	7	8.86
Misleading interpretation	Title claims or suggests a beneficial effect of the experimental intervention not supported by the findings	4	5.06
	Inadequate focus on P-value instead of magnitude of the effect estimates for harm or efficacy outcome	28	35.44
	Focus on relative effect when the absolute effect is small	6	7.59
	Conclusion claiming equivalence or comparable effectiveness for nonstatistically significant results with a wide confidence interval	4	5.06
	Conclusion formulating recommendations for clinical practice not supported by the findings	28	35.44
	Conclusion claiming safety based on nonstatistically significant results with a wide confidence interval	3	3.80
	Conclusion claiming the beneficial effect of the experimental treatment despite high risk of bias in primary studies	67	84.81
	Conclusion claiming the beneficial effect of the experimental treatment despite reporting bias	6	7.59
	Conclusion claiming the beneficial effect of the experimental treatment despite high heterogeneity	9	11.39
	Ignoring that the review included different study design (e.g., controlled trial or observational studies)	39	49.37
Inappropriate extrapolation	Conclusion extrapolating the review's findings to a different population or setting	17	21.52
	Conclusion extrapolating the review's findings to a different intervention (i.e., claiming efficacy of one specific intervention although the review covers a class of several interventions)	2	2.53
	Conclusion extrapolating the review's findings from a surrogate marker or a specific outcome to the global improvement of the disease	1	1.27

### Statistical analysis

Descriptive statistics, including frequency and percentage or the means and standard deviations, were utilized to characterize the frequency of each type of spin and the overall spin score. Furthermore, the five most common types of spin and the five most severe types of spin as defined by Yavchitz et al. [27] were assessed further by correlating spin type with the level of evidence and the year of publication. Spearman’s rank-order correlation was used to compare categorical variables (spin type with level of evidence and year of publication). The five most severe types included “conclusion formulating recommendations for clinical practice not supported by the findings,” “title claims or suggests a beneficial effect of the experimental intervention not supported by the findings,” “selective reporting of or overemphasis on efficacy outcomes favoring the beneficial effect of the experimental intervention,” “conclusion claiming safety based on non-statistically significant results with a wide confidence interval,” and “conclusion claiming the beneficial effect of the experimental treatment despite high risk of bias in primary studies.” Statistical analyses were performed using SPSS version 28.0 (IBM, Armonk, New York, USA).

## Results

### General

**Fig. 2:**
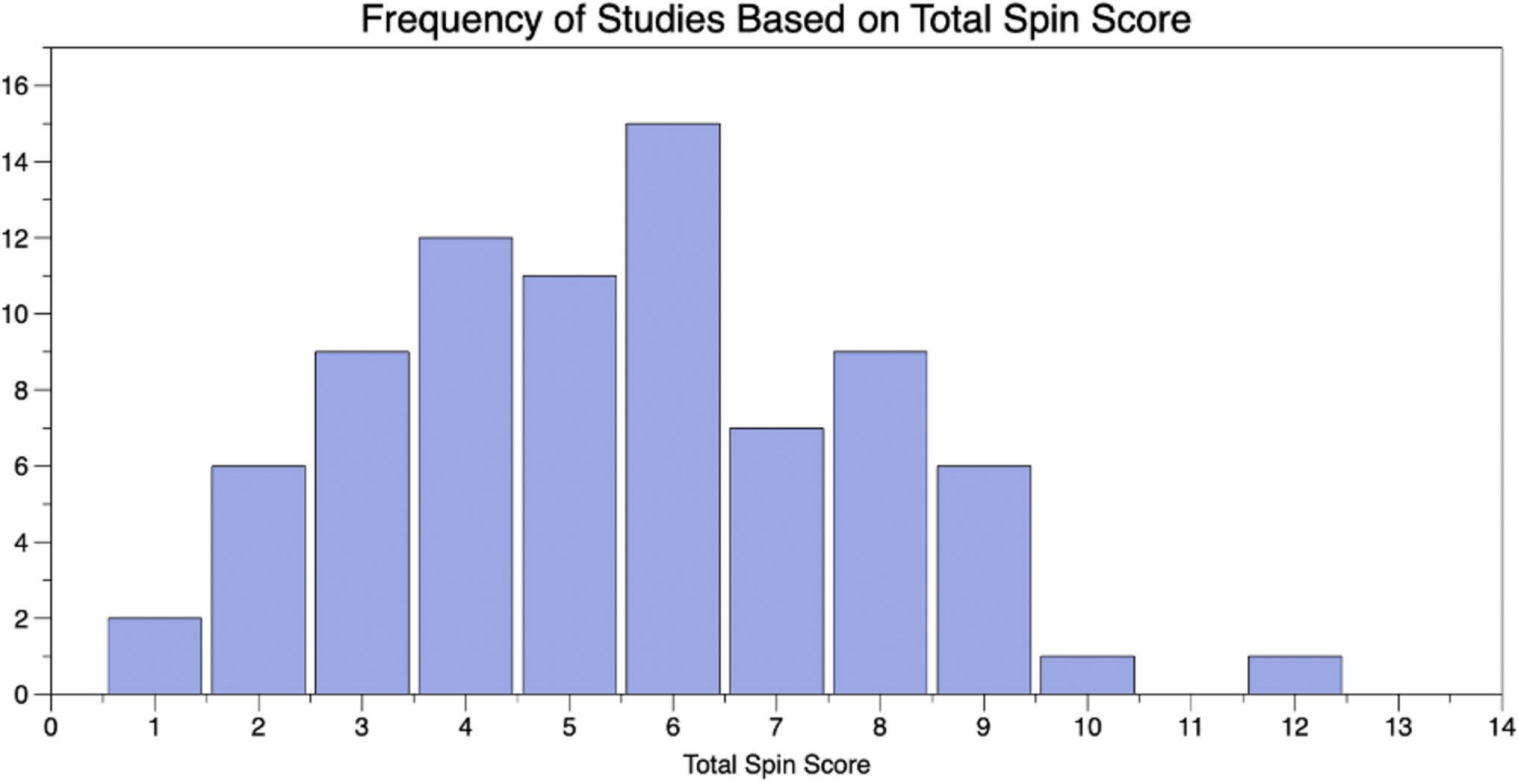
Frequency histogram of total spin score

In all 79 systematic reviews and meta-analyses assessed, at least one form of spin was identified. The average spin score per paper was 5.5 ± 2.3 (range 1–12) (Fig. 2). The most common type of spin was “conclusion claiming the beneficial effect of the experimental treatment despite high risk of bias in primary studies,” which was observed in 67 of 79 studies (84.8%). The next most common types of spin included “failure to report a wide confidence interval of estimates” (57/79, 72.2%), “authors hide or do not present any conflicts of interest” (46/79, 58.2%), “ignoring that the review included different study design” (39/79, 49.4%), and “failure to report the number of studies/patients actually contributing to the analysis for main outcomes” (39/79, 49.4%). The least common type of spin was “conclusion extrapolating the review's findings from a surrogate marker or a specific outcome to the global improvement of the disease” (1/79, 1.3%) (Table 1).

The frequency of the five most severe types of spin evaluated were: 84.8% (67/79) included “conclusion claiming the beneficial effect of the experimental treatment despite high risk of bias in primary studies,” 36.7% (29/79) of abstracts included the spin type “conclusion formulating recommendations for clinical practice not supported by the findings,” 36.7% (29/79) included “selective reporting of or overemphasis on efficacy outcomes favoring the beneficial effect of the experimental intervention,” 5.1% (4/79) included “title claims or suggests a beneficial effect of the experimental intervention not supported by the findings,” and 3.8% (3/79) included “conclusion claiming safety based on non-statistically significant results with a wide confidence interval.”

The manuscripts reviewed were most frequently published in 2021 (22/79, 27.8%) or 2022 (14/70, 17.7%). They most frequently contained level 1 evidence or exclusively included randomized controlled trials (RCTs) (62/79, 78.5%). The funding source was not listed for 37 of the included studies (46.8%), while 19 (24.1%) received funding and 23 (29.1%) did not. The majority of included studies mentioned PRISMA guidelines (59/79, 74.7%), while only 18 (22.8%) registered a protocol prior to completion of the meta-analysis or systematic review (Table 2). Of those manuscripts identifiable on Scopus CiteScore™ (66/79, 83.5%), the average score was 3.4 ± 3.6 (range 0–17.11).

**Table 2:** Descriptive frequencies for paper characteristics

		*N*	%
Publication date	2013	1	1.3
	2014	4	5.1
	2015	4	5.1
	2016	5	6.3
	2017	7	8.9
	2018	5	6.3
	2019	7	8.9
	2020	10	12.7
	2021	22	27.8
	2022	14	17.7
Level of evidence	1	62	78.5
	2	10	12.7
	3	3	3.8
	4	3	3.8
Funding	Not funded	23	29.1
	Funded	19	24.1
	Not listed	37	46.8
PRISMA mentioned	No	20	25.3
	Yes	59	74.7
Registered protocol	No	61	77.2
	Yes	18	22.8

### Associations between study characteristics and spin types

Upon evaluation of the five most common types of spin, there was a correlation between the spin type “ignoring that the review included different study designs” and the level of evidence (rho: 0.269, *P-*value: 0.017). Similarly, the only correlation between the presence of these spin types and the year of publication was a correlation between the spin type “authors hide or do not present any conflict of interest” and an older publication year (rho: − 0.252, *P *value: 0.025) (Table 3).

**Table 3:** Correlation between level of evidence and the top five most common spin types

	Spin types	Reporting spin		Association with level of evidence	Association with year of publication
		*N*	%	*P* value	
Most common spin	Bias	67	84.8	0.688	0.453
	Confidence intervals	57	72.2	0.507	0.578
	Ignoring review included different	39	49.4	0.066	0.29
	Failure to report number of patients	39	49.4	0.096	0.711
	Author conflict of interest	46	58.2	0.486	0.003
Most severe spin	Clinical practice recommendations not supported by findings	29	36.71	0.605	0.938
	Title suggests beneficial effects not supported by findings	4	5.06	0.105	0.959
	Selective reporting favoring beneficial effect	29	36.71	0.177	0.895
	Conclusion claiming safety	3	3.80	0.041	0.783
	Bias	67	84.81	0.688	0.453

Upon evaluation of the five most severe types of spin, there were no correlations between spin type and level of evidence, or the spin type and the year of publication (*P *value > 0.05). (Table 3).

## Discussion

The present study established that spin favoring the utilization of PRP injections is highly prevalent in abstracts of systematic reviews and meta-analyses for the treatment of knee osteoarthritis. We demonstrated high frequency of spin, particularly those relating to misleading reporting or misleading data interpretation, high frequency of lack of identification of funding sources, limited protocol registration, and relatively low Scopus CiteScore™. As the first study of its kind, we noted consistent presentation of inaccurate information distributed via the abstract of manuscripts related to PRP [23, 31–36]. This is of particular concern given the increasing patient awareness of PRP as a therapeutic option in the treatment of knee osteoarthritis, the increased utilization of PRP among orthopedic surgeons, and the well-documented reality that physicians often make clinical decisions based solely on abstracts [7, 16, 21, 37, 38]. These findings indicate appropriately powered and blinded RCTs with results presented in the absence of spin are necessary to (1) better evaluate the utility of PRP injections for knee osteoarthritis and (2) better counsel patients regarding expectations of this therapy [24, 25, 27].

The most frequently reported spin types included “conclusion claiming the beneficial effect of the experimental treatment despite high risk of bias in primary studies” (84.8%), “failure to report a wide confidence interval of estimates” (72.2%), “authors hide or do not present any conflicts of interest” (58.2%), “ignoring that the review included different study design” (49.4%), and “failure to report the number of studies/patients actually contributing to the analysis for main outcomes” (49.4%). Furthermore, we identified at least one type of spin in 100% of assessed abstracts. In a spin assessment of systematic reviews and meta-analyses evaluating superior capsular reconstruction in the shoulder literature, Kim et al. [23] utilized a similar methodology in an evaluation of superior capsular release and reported a high frequency of “the conclusion claims the beneficial effect of the experimental treatment despite a high risk of bias in primary studies” at 65%. They similarly found 100% of included studies had at least one type of spin. In a study of PRP utilized for the treatment of orthopedic injury of the hand, foot and ankle, spine, hip, and knee, Reddy et al. [36] reported only 28.4% of included studies reported at least one type of spin. The rates of spin in the present study were similar to these previous reports, indicating that the abstracts included in systematic reviews and meta-analyses of PRP used for treatment of knee osteoarthritis have the potential to include misleading language.

Outside of the orthopedic literature, however, the primary sources of spin differ and the overall frequency of spin appears to be substantially lower. For example, in an assessment of systematic reviews and meta-analyses focusing on percutaneous coronary intervention, Cole et al. [33] reported only 22.1% of included studies had at least one type of bias. Their most frequent form of spin, “selective reporting of, or overemphasis on efficacy outcomes or analysis favoring the beneficial effect of the experimental intervention,” was only present in 14.8% of included abstracts. Demla et al. [34] applied a similar spin assessment to systematic reviews and meta-analyses evaluating cataract therapies and found only 30.0% of included studies had at least one form of spin. Their highest frequency was also in the category “selective reporting of, or overemphasis on efficacy outcomes or analysis favoring the beneficial effect of the experimental intervention” (18.2%), which is also substantially lower than those noted in the present study, or that by Kim et al. In fact, these frequencies (14.8% and 18.2%) more closely resemble our 10th (21.5%) and 11th (12.7%) most common types of spin. Although a relative weighting is not a component of the spin scoring system, we additionally reported most severe types of spin recorded. To our knowledge, no other study to date implementing spin assessment has done this. While additional studies are needed to evaluate spin in other orthopedic procedures and topics, this represents concerning data, suggesting that systematic reviews and meta-analyses in the orthopedic literature may be vulnerable to distorted data reporting that over or understates effects with insufficient evidence when compared to other fields.

Although PRP injections in the treatment of knee osteoarthritis are increasingly utilized, results such as these should give pause to orthopedic surgeons when evaluating the quality of data available to support their use. We acknowledge that high-quality data exist supporting the therapeutic effects of PRP when utilized for knee osteoarthritis [39–42]; however, the overall body of literature shows mixed effects, particularly with respect to functional, radiographic, and pain outcomes [9, 43–46]. Furthermore, given that PRP injections are not currently approved by the United States Food and Drug Association (FDA), many patients must pay for these procedures out of pocket. The average reported costs may be in excess of $714 per treatment, and some physicians utilize multiple injections for a given treatment course [43]. Moreover, the American Association of Hip and Knee Surgeons statement does not currently recommend the use of PRP due to the lack of beneficence, the high-cost, and potential safety issues [9]. The potential negative implications of elevated spin and distorted reporting in systematic reviews and meta-analyses may result in ongoing misinformation and misinterpretation of data related to these procedures. This evaluation similarly calls for increased high-quality assessments and a heightened awareness of spin by providers and journals as it pertains to distribution of information, and its impact on patient care and outcomes.

There are several limitations and strengths of this evaluation. Many of the included abstracts were published in journals in which there are word count limitations. This indicates that some of the included studies may have unintentionally spun findings due to an inability to include the level of detail needed to provide appropriate context and reduce bias. However, there is a strong impetus for abstracts to be both accurate and non-distorted relative to the manuscript as many individuals reading the abstract may not have access to the full-text, nor the time to read the entire manuscript. Therefore, the present evaluation provides substantial value to understand how an orthopedic surgeon may interpret the body of literature describing PRP injections for knee osteoarthritis. Moreover, our findings should caution orthopedic surgeons considering PRP injections given spin in abstracts can lead to misinterpretation of results, and identification of favorable results even when studies lack significant findings [25, 32, 47]. Furthermore, this study is inherently limited by the spin tool employed. However, Yavchitz et al. [27] performed validation of their assessment tool, and it has been utilized a number of times in the published literature. Aside from these limitations, we utilized a validated tool, included a large sample of systematic reviews and meta-analyses, and had a high degree of adherence to an established assessment protocol. Moreover, we demonstrated novel and concerning findings that highlight the need for improved reporting in orthopedic systematic reviews and meta-analyses assessing the utilization of PRP injections for knee osteoarthritis. Ideally, orthopedic surgeons should pause prior to indicating patients for this treatment, to consider the quality of the evidence supporting its utilization particularly in light of the long-term outcomes and associated costs.

## Conclusion

Spin favoring PRP is highly prevalent in systematic reviews and meta-analyses that assess the use of PRP for the treatment of knee osteoarthritis. This distortion of information identifies a need for (1) high-quality RCTs and (2) reduction of spin in the abstracts of future literature to accurately distribute information regarding PRP for the treatment of knee osteoarthritis.

## Supplementary Information

Below is the link to the electronic supplementary material.

Supplementary file1 (DOCX 20 kb)

## Data Availability

No datasets were generated or analyzed during the current study.
