# Supplementary material for: Spin and reporting bias in the use of platelet-rich plasma for the treatment of knee osteoarthritis
Source: Eur J Orthop Surg Traumatol. 2025 Aug 12;35(1):348. doi: 10.1007/s00590-025-04353-x (PMC12343690; doi:10.1007/s00590-025-04353-x)
Supplement: Supplementary file 1 — Supplementary file1 (DOCX 20 kb) [file 590_2025_4353_MOESM1_ESM.docx]

| **First Author and Publication Year** | **Title** |
| --- | --- |
| Anil 2021 | The efficacy of intra-articular injections in the treatment of knee osteoarthritis: A network meta-analysis of randomized controlled trials[[48]](https://paperpile.com/c/BKHnFV/QeK1) |
| Anitua 2014 | Efficacy and safety of plasma rich in growth factors intra-articular infiltrations in the treatment of knee osteoarthritis[[49]](https://paperpile.com/c/BKHnFV/erbS) |
| Anzillotti 2022 | Injection of biologic agents for treating severe knee osteoarthritis: is there a chance for a good outcome? A systematic review of clinical evidence[[50]](https://paperpile.com/c/BKHnFV/5mfI) |
| Aw 2021 | Comparing the efficacy of dual Platelet-Rich Plasma (PRP) and Hyaluronic Acid (HA) therapy with PRP-alone therapy in the treatment of knee osteoarthritis: a systematic review and meta-analysis[[51]](https://paperpile.com/c/BKHnFV/xwhE) |
| Baria 2022 | Treating Knee Osteoarthritis With Platelet-Rich Plasma and Hyaluronic Acid Combination Therapy: A Systematic Review[[52]](https://paperpile.com/c/BKHnFV/gIp8) |
| Berkani 2022 | Time to Total Knee Arthroplasty after Intra-Articular Hyaluronic Acid or Platelet-Rich Plasma Injections: A Systematic Literature Review and Meta-Analysis[[53]](https://paperpile.com/c/BKHnFV/HD05) |
| Betzler 2021 | Intra-articular injection of orthobiologics in patients undergoing high tibial osteotomy for knee osteoarthritis is safe and effective - a systematic review[[54]](https://paperpile.com/c/BKHnFV/HJRd) |
| Campbell 2015 | Does Intra-articular Platelet-Rich Plasma Injection Provide Clinically Superior Outcomes Compared With Other Therapies in the Treatment of Knee Osteoarthritis? A Systematic Review of Overlapping Meta-analyses[[20]](https://paperpile.com/c/BKHnFV/VUkFQ) |
| Chang 2014 | Comparative effectiveness of platelet-rich plasma injections for treating knee joint cartilage degenerative pathology: a systematic review and meta-analysis[[55]](https://paperpile.com/c/BKHnFV/H4OJ) |
| Charlesworth 2019 | Osteoarthritis- a systematic review of long-term safety implications for osteoarthritis of the knee[[56]](https://paperpile.com/c/BKHnFV/7oMh) |
| Chen 2019 | Intra-articular platelet-rich plasma injection for knee osteoarthritis: a summary of meta-analyses[[57]](https://paperpile.com/c/BKHnFV/jOLz) |
| Chen 2020 | Platelet-rich plasma versus hyaluronic acid in the treatment of knee osteoarthritis: A meta-analysis[[58]](https://paperpile.com/c/BKHnFV/GyG7) |
| Chou 2021 | Efficacy of different platelet-rich plasma injections in the treatment of mild-moderate knee osteoarthritis: A systematic review and meta-analysis[[59]](https://paperpile.com/c/BKHnFV/sgxq) |
| Costa 2022 | How Does Platelet-Rich Plasma Compare Clinically to Other Therapies in the Treatment of Knee Osteoarthritis? A Systematic Review and Meta-analysis[[60]](https://paperpile.com/c/BKHnFV/BfAT) |
| Dai 2017 | Efficacy of Platelet-Rich Plasma in the Treatment of Knee Osteoarthritis: A Meta-analysis of Randomized Controlled Trials[[61]](https://paperpile.com/c/BKHnFV/2qiC) |
| Delanois 2019 | Biologic Therapies for the Treatment of Knee Osteoarthritis[[6]](https://paperpile.com/c/BKHnFV/5fBXo) |
| Delanois 2022 | Biologic Therapies for the Treatment of Knee Osteoarthritis: An Updated Systematic Review[[62]](https://paperpile.com/c/BKHnFV/stlS) |
| Di 2018 | Is local platelet-rich plasma injection clinically superior to hyaluronic acid for treatment of knee osteoarthritis? A systematic review of randomized controlled trials[[63]](https://paperpile.com/c/BKHnFV/IYLh) |
| Dold 2014 | Platelet-rich plasma in the management of articular cartilage pathology: a systematic review[[64]](https://paperpile.com/c/BKHnFV/XtUC) |
| Dong 2021 | The effects of platelet-rich plasma injection in knee and hip osteoarthritis: a meta-analysis of randomized controlled trials[[65]](https://paperpile.com/c/BKHnFV/nY1c) |
| El-beltagy 2021 | Intra-Articular Injections of Platelet-Rich Plasma versus Hyaluronic Acid in Treatment of Knee Osteoarthritis (Systematic Review and Meta-analysis)[[66]](https://paperpile.com/c/BKHnFV/INOZ) |
| Filardo 2021 | PRP Injections for the Treatment of Knee Osteoarthritis: A Meta-Analysis of Randomized Controlled Trials[[67]](https://paperpile.com/c/BKHnFV/FlRk) |
| Gilat 2021 | Hyaluronic acid and platelet-rich plasma for the management of knee osteoarthritis[[68]](https://paperpile.com/c/BKHnFV/lMGw) |
| Gong 2021 | Clinical therapy of platelet-rich plasma vs hyaluronic acid injections in patients with knee osteoarthritis: A systematic review and meta-analysis of randomized double-blind controlled trials[[69]](https://paperpile.com/c/BKHnFV/zKp2) |
| Han 2021 | Intra-Articular Injections of Hyaluronic Acid or Steroids Associated With Better Outcomes Than Platelet-Rich Plasma, Adipose Mesenchymal Stromal Cells, or Placebo in Knee Osteoarthritis: A Network Meta-analysis[[70]](https://paperpile.com/c/BKHnFV/PSdc) |
| Han 2019 | Meta-analysis Comparing Platelet-Rich Plasma vs Hyaluronic Acid Injection in Patients with Knee Osteoarthritis[[71]](https://paperpile.com/c/BKHnFV/S1aR) |
| Hegazy 2019 | Meaningful effectiveness of platelet-rich plasma (PRP) in treating patients with osteoarthritis of the knee: Meta-analysis and review[[72]](https://paperpile.com/c/BKHnFV/9mTe) |
| Hohmann 2020 | Is platelet-rich plasma effective for the treatment of knee osteoarthritis? A systematic review and meta-analysis of level 1 and 2 randomized controlled trials[[73]](https://paperpile.com/c/BKHnFV/Lfto) |
| Hong 2021 | Efficacy and Safety of Intra-Articular Platelet-Rich Plasma in Osteoarthritis Knee: A Systematic Review and Meta-Analysis[[39]](https://paperpile.com/c/BKHnFV/zv2lo) |
| Johal 2019 | Impact of Platelet-Rich Plasma Use on Pain in Orthopaedic Surgery: A Systematic Review and Meta-analysis[[74]](https://paperpile.com/c/BKHnFV/VAHf) |
| Kanchanatawan 2016 | Short-term outcomes of platelet-rich plasma injection for treatment of osteoarthritis of the knee[[75]](https://paperpile.com/c/BKHnFV/xOM7) |
| Karasavvidis 2021 | Platelet-Rich Plasma Combined With Hyaluronic Acid Improves Pain and Function Compared With Hyaluronic Acid Alone in Knee Osteoarthritis: A Systematic Review and Meta-analysis[[76]](https://paperpile.com/c/BKHnFV/bLb9) |
| Khoshbin 2013 | The efficacy of platelet-rich plasma in the treatment of symptomatic knee osteoarthritis: a systematic review with quantitative synthesis[[77]](https://paperpile.com/c/BKHnFV/1q8G) |
| Kim 2022 | Are leukocyte-poor or multiple injections of platelet-rich plasma more effective than hyaluronic acid for knee osteoarthritis? A systematic review and meta-analysis of randomized controlled trials[[78]](https://paperpile.com/c/BKHnFV/WGDH) |
| Kim 2021 | Adverse Reactions and Clinical Outcomes for Leukocyte-Poor Versus Leukocyte-Rich Platelet-Rich Plasma in Knee Osteoarthritis: A Systematic Review and Meta-analysis[[79]](https://paperpile.com/c/BKHnFV/Bkdm) |
| Knop 2016 | Platelet-rich plasma for osteoarthritis treatment[[80]](https://paperpile.com/c/BKHnFV/4ymf) |
| Lai 2015 | Use of Platelet-Rich Plasma in Intra-Articular Knee Injections for Osteoarthritis: A Systematic Review[[81]](https://paperpile.com/c/BKHnFV/k0Ve) |
| Laudy 2015 | Efficacy of platelet-rich plasma injections in osteoarthritis of the knee: a systematic review and meta-analysis[[18]](https://paperpile.com/c/BKHnFV/belDk) |
| Laver 2017 | PRP for Degenerative Cartilage Disease: A Systematic Review of Clinical Studies[[13]](https://paperpile.com/c/BKHnFV/g0JB) |
| Li 2020 | Comparative efficacy of treatments for patients with knee osteoarthritis: a network meta-analysis[[82]](https://paperpile.com/c/BKHnFV/q833) |
| Li 2022 | The application of platelet-rich plasma in the treatment of knee osteoarthritis: A literature review[[83]](https://paperpile.com/c/BKHnFV/YwRW) |
| Lin 2022 | Comparing the efficacy of different intra-articular injections for knee osteoarthritis: A network analysis[[84]](https://paperpile.com/c/BKHnFV/R0Kq) |
| Luo 2020 | How to Choose Platelet-Rich Plasma or Hyaluronic Acid for the Treatment of Knee Osteoarthritis in Overweight or Obese Patients: A Meta-Analysis[[85]](https://paperpile.com/c/BKHnFV/YgR9) |
| McLarnon 2021 | Intra-articular platelet-rich plasma injections versus intra-articular corticosteroid injections for symptomatic management of knee osteoarthritis: systematic review and meta-analysis[[86]](https://paperpile.com/c/BKHnFV/mi39) |
| Meheux 2016 | Efficacy of Intra-articular Platelet-Rich Plasma Injections in Knee Osteoarthritis: A Systematic Review[[19]](https://paperpile.com/c/BKHnFV/vS5Jf) |
| Migliorini 2021 | Comparison between intra-articular infiltrations of placebo, steroids, hyaluronic and PRP for knee osteoarthritis: a Bayesian network meta-analysis[[87]](https://paperpile.com/c/BKHnFV/sEAG) |
| Mojica 2022 | Estimated Time to Maximum Medical Improvement of Intra-articular Injections in the Treatment of Knee Osteoarthritis-A Systematic Review[[88]](https://paperpile.com/c/BKHnFV/hiI2) |
| Muchedzi 2017 | A systematic review of the use of platelet rich plasma in patients with knee osteoarthritis (OA) and its effect on patient outcomes following surgical interventions for knee OA[[89]](https://paperpile.com/c/BKHnFV/0mtS) |
| Muchedzi 2018 | A systematic review of the effects of platelet rich plasma on outcomes for patients with knee osteoarthritis and following total knee arthroplasty[[90]](https://paperpile.com/c/BKHnFV/Kuq6) |
| Naja 2021 | Comparative effectiveness of nonsurgical interventions in the treatment of patients with knee osteoarthritis: A PRISMA-compliant systematic review and network meta-analysis[[91]](https://paperpile.com/c/BKHnFV/9tNG) |
| Nie 2021 | Effectiveness of Platelet-Rich Plasma in the Treatment of Knee Osteoarthritis: A Meta-analysis of Randomized Controlled Clinical Trials[[92]](https://paperpile.com/c/BKHnFV/GE04) |
| Peng 2022 | Intra-Articular Leukocyte-Rich Platelet-Rich Plasma versus Intra-Articular Hyaluronic Acid in the Treatment of Knee Osteoarthritis: A Meta-Analysis of 14 Randomized Controlled Trials[[93]](https://paperpile.com/c/BKHnFV/IlVy) |
| Phillips 2020 | Differentiating factors of intra-articular injectables have a meaningful impact on knee osteoarthritis outcomes: a network meta-analysis[[94]](https://paperpile.com/c/BKHnFV/ZyKH) |
| Raeissadat 2021 | Effectiveness of intra-articular autologous-conditioned serum injection in knee osteoarthritis: A meta-analysis study[[95]](https://paperpile.com/c/BKHnFV/cpP7) |
| Ren 2020 | Role of platelet-rich plasma in the treatment of osteoarthritis: a meta-analysis[[96]](https://paperpile.com/c/BKHnFV/s9m2) |
| Riboh 2016 | Effect of Leukocyte Concentration on the Efficacy of Platelet-Rich Plasma in the Treatment of Knee Osteoarthritis[[97]](https://paperpile.com/c/BKHnFV/KPfJ) |
| Rodriguez-Garcia 2021 | Efficacy and safety of intra-articular therapies in rheumatic and musculoskeletal diseases: an overview of systematic reviews[[98]](https://paperpile.com/c/BKHnFV/2nUk) |
| Sadabad 2016 | Efficacy of Platelet-Rich Plasma versus Hyaluronic Acid for treatment of Knee Osteoarthritis: A systematic review and meta-analysis[[99]](https://paperpile.com/c/BKHnFV/sUGh) |
| Sax 2022 | The Efficacy of Platelet-Rich Plasma for the Treatment of Knee Osteoarthritis Symptoms and Structural Changes: A Systematic Review and Meta-Analysis[[100]](https://paperpile.com/c/BKHnFV/jaGU) |
| Shen 2017 | The temporal effect of platelet-rich plasma on pain and physical function in the treatment of knee osteoarthritis: systematic review and meta-analysis of randomized controlled trials[[101]](https://paperpile.com/c/BKHnFV/q6wv) |
| Shi 2017 | Biologic injections for osteoarthritis and articular cartilage damage: can we modify disease?[[102]](https://paperpile.com/c/BKHnFV/XlXA) |
| Singh 2022 | Relative Efficacy of Intra-articular Injections in the Treatment of Knee Osteoarthritis: A Systematic Review and Network Meta-analysis[[103]](https://paperpile.com/c/BKHnFV/8AA3) |
| Souzdalnitski 2015 | Platelet-rich plasma injections for knee osteoarthritis: Systematic review of duration of clinical benefit[[104]](https://paperpile.com/c/BKHnFV/gkVc) |
| Tan 2021 | Platelet-Rich Plasma Versus Hyaluronic Acid in the Treatment of Knee Osteoarthritis: A Meta-analysis of 26 Randomized Controlled Trials[[105]](https://paperpile.com/c/BKHnFV/49SN) |
| Tang 2020 | Platelet-rich plasma versus hyaluronic acid in the treatment of knee osteoarthritis: a meta-analysis[[106]](https://paperpile.com/c/BKHnFV/GYr9) |
| Tietze 2014 | The effects of platelet-rich plasma in the treatment of large-joint osteoarthritis: a systematic review[[107]](https://paperpile.com/c/BKHnFV/P7sN) |
| Trams 2020 | The Clinical Use of Platelet-Rich Plasma in Knee Disorders and Surgery-A Systematic Review and Meta-Analysis[[108]](https://paperpile.com/c/BKHnFV/5RhF) |
| Trams 2022 | Role of Platelets in Osteoarthritis-Updated Systematic Review and Meta-Analysis on the Role of Platelet-Rich Plasma in Osteoarthritis[[10]](https://paperpile.com/c/BKHnFV/EawY8) |
| Ubilla 2018 | Is platelet-rich plasma effective for osteoarthritis?[[109]](https://paperpile.com/c/BKHnFV/8r4u) |
| Vannabouathong 2018 | Nonoperative Treatments for Knee Osteoarthritis: An Evaluation of Treatment Characteristics and the Intra-Articular Placebo Effect: A Systematic Review[[110]](https://paperpile.com/c/BKHnFV/eNae) |
| Vilchez-Cavazos 2022 | The use of platelet-rich plasma in studies with early knee osteoarthritis versus advanced stages of the disease: a systematic review and meta-analysis of 31 randomized clinical trials[[111]](https://paperpile.com/c/BKHnFV/4NXt) |
| Vilchez-Cavazos 2019 | Comparison of the Clinical Effectiveness of Single Versus Multiple Injections of Platelet-Rich Plasma in the Treatment of Knee Osteoarthritis: A Systematic Review and Meta-analysis[[112]](https://paperpile.com/c/BKHnFV/9Rq9) |
| Wei 2021 | Platelet-Rich Plasma and Stem Cell Injections in the Treatment of Arthritis of the Knee[[113]](https://paperpile.com/c/BKHnFV/vzOg) |
| Wu 2020 | Platelet-rich plasma versus hyaluronic acid in knee osteoarthritis: A meta-analysis with the consistent ratio of injection[[114]](https://paperpile.com/c/BKHnFV/Q7A5) |
| Xing 2017 | Intra-articular platelet-rich plasma injections for knee osteoarthritis: An overview of systematic reviews and risk of bias considerations[[115]](https://paperpile.com/c/BKHnFV/dkPz) |
| Xu 2017 | Efficacy of Platelet-Rich Plasma in Pain and Self-Report Function in Knee Osteoarthritis: A Best-Evidence Synthesis[[116]](https://paperpile.com/c/BKHnFV/bqgV) |
| Zhang 2018 | Intra-articular platelet-rich plasma versus hyaluronic acid in the treatment of knee osteoarthritis: a meta-analysis[[117]](https://paperpile.com/c/BKHnFV/L3gN) |
| Zhao 2021 | Intra-Articular Injections of Platelet-Rich Plasma, Adipose Mesenchymal Stem Cells, and Bone Marrow Mesenchymal Stem Cells Associated With Better Outcomes Than Hyaluronic Acid and Saline in Knee Osteoarthritis: A Systematic Review and Network Meta-analysis[[118]](https://paperpile.com/c/BKHnFV/k3kT) |
| Zhao 2020 | Effects and safety of the combination of platelet-rich plasma (PRP) and hyaluronic acid (HA) in the treatment of knee osteoarthritis: a systematic review and meta-analysis[[17]](https://paperpile.com/c/BKHnFV/Nt8t0) |

**Supplemental Table 1:** Abstracts eligible and included in data extraction and statistical analyses.
